# An Evaluation of Primary Studies Published in Predatory Journals Included in Systematic Reviews From High-Impact Dermatology Journals: Cross-sectional Study

**DOI:** 10.2196/39365

**Published:** 2022-09-14

**Authors:** Ryan Ottwell, Brooke Hightower, Olivia Failla, Kelsey Snider, Adam Corcoran, Micah Hartwell, Matt Vassar

**Affiliations:** 1 Department of Dermatology St Jospeh Mercy Ypsilanti, MI United States; 2 Oklahoma State University Center for Health Sciences Tulsa, OK United States

**Keywords:** predatory journals, systematic review, general dermatology, dermatology, publishing, publications, journals, scientific communication, data, quality, meta-analysis, peer review, primary studies, research, evidence synthesis, articles

## Abstract

**Background:**

Predatory publishing is a deceptive form of publishing that uses unethical business practices, minimal to no peer review processes, or limited editorial oversight to publish articles. It may be problematic to our highest standard of scientific evidence—systematic reviews—through the inclusion of poor-quality and unusable data, which could mislead results, challenge outcomes, and undermine confidence. Thus, there is a growing concern surrounding the effects predatory publishing may have on scientific research and clinical decision-making.

**Objective:**

The objective of this study was to evaluate whether systematic reviews published in top dermatology journals contain primary studies published in suspected predatory journals (SPJs).

**Methods:**

We searched PubMed for systematic reviews published in the top five dermatology journals (determined by 5-year h-indices) between January 1, 2019, and May 24, 2021. Primary studies were extracted from each systematic review, and the publishing journal of these primary studies was cross-referenced using Beall’s List and the Directory of Open Access Journals. Screening and data extraction were performed in a masked, duplicate fashion. We performed chi-square tests to determine possible associations between a systematic review’s inclusion of a primary study published in a SPJ and particular study characteristics.

**Results:**

Our randomized sample included 100 systematic reviews, of which 31 (31%) were found to contain a primary study published in a SPJ. Of the top five dermatology journals, the *Journal of the American Academy of Dermatology* had the most systematic reviews containing a primary study published in an SPJ. Systematic reviews containing a meta-analysis or registered protocol were significantly less likely to contain a primary study published in a SPJ. No statistically significant associations were found between other study characteristics.

**Conclusions:**

Studies published in SPJs are commonly included as primary studies in systematic reviews published in high-impact dermatology journals. Future research is needed to investigate the effects of including suspected predatory publications in scientific research.

## Introduction

Predatory publishing is described as a “nebulous concept of research journal publishers who use unethical business practices, minimal or no peer review, or limited editorial oversight to publish articles that are below a minimally accepted standard of quality” [1]. Increasing rates of predatory publishing are accompanied by an equally growing concern surrounding their threat to evidence synthesis and decision-making [1,2]. Predatory publishing can be problematic to our highest standard of scientific evidence—systematic reviews (SRs)—through the inclusion of poor-quality and unusable data, which could mislead results, challenge outcomes, and undermine confidence due to suspected predatory journals (SPJs) having a less rigorous peer review process.[3] Evidence is lacking as to whether studies published in SPJs are frequently included as primary studies in SRs; therefore, we aimed to evaluate whether SRs published in top dermatology journals contain primary studies published in SPJ.

## Methods

We searched PubMed (using the Advanced Search filters) for SRs published in the top five dermatology journals (determined by 5-year h-indices) between January 01, 2019, and May 24, 2021. The returned SRs (N=339) were downloaded as a comma-separated values file. We randomized the returns and selected the first 100 articles to examine. Primary studies were extracted from each systematic review, and the publishing journal of these primary studies was cross-referenced using Beall’s List (archived and updated versions [4]) and the Directory of Open Access Journals (DOAJ) [5], both widely used and publicly available databases of suspected predatory or questionable journals. To determine if certain study characteristics were associated with the inclusion of SPJs, the following characteristics were extracted: (1) whether the SR received funding; (2) whether the SR had a registered protocol; (3) whether the SR included randomized controlled trials, nonrandomized studies of interventions, or both as primary studies; (4) the year the SR was published; and (5) the databases the SR searched for primary studies, to determine if certain study characteristics were associated with the inclusion of SPJs. Screening and data extraction were performed in a masked, duplicate fashion by authors BH and KS, in accordance with best practices [6]. We performed chi-square tests to determine possible associations between an SR’s inclusion of a primary study published in an SPJ and particular study characteristics.

This study did not use human subjects and thus did not require institutional review board oversight.

## Results

Our randomized sample included 100 SRs, of which 31 (31%) SRs were found to contain a primary study published in an SPJ. A total of 53 primary studies were published across 22 unique SPJs. Of the top five dermatology journals, the *Journal of the American Academy of Dermatology* had the most SRs containing a primary study published in an SPJ (Table 1). The majority of suspected predatory publications (28/55, 51%) were published in the *Indian Journal of Dermatology, Venereology, and Leprology*. SRs that contained a meta-analysis were significantly less likely to contain a primary study published in an SPJ (*P*=.002; Table 1). Additionally, SRs that had a registered protocol were less likely to contain a primary study published in an SPJ (*P*=.02). No statistically significant associations were found between journals, year of publication, included primary study types (eg, randomized controlled trials, nonrandomized studies of interventions, or both), funding, or databases included in the SR search.

**Table 1:** Characteristics of systematic reviews with and without primary studies published in predatory journals (N=100).

Study characteristics		Contains a primary study published in a suspected predatory journal, n (%)				Chi-square (*df*)		*P* value
		No	Yes	Total				
**Journal**						3.69 (4,1)		.45
	*Journal of the American Academy of Dermatology*	26 (26)	17 (17)	43 (43)				
	*British Journal of Dermatology*	12 (12)	4 (4)	16 (16)				
	*Journal of Investigative Dermatology*	1 (1)	0 (0)	1 (1)				
	*Journal of the European Academy of Dermatology and Venereology*	19 (19)	8 (8)	27 (27)				
	*Jama Dermatology*	11 (11)	2 (2)	13 (13)				
**Year of publication**						0.64 (2,1)		.07
	2019	24 (24)	13 (13)	37 (37)				
	2020	30 (30)	11 (11)	41 (41)				
	2021	15 (15)	7 (7)	22 (22)				
**Systematic review contained a meta-analysis**						9.38 (1,1)		.002
	No	20 (20)	19 (19)	39 (39)				
	Yes	49 (49)	12 (12)	61 (61)				
**Study received funding**						2.69 (1,1)		.10
	No	47 (47)	26 (26)	73 (73)				
	Yes	22 (22)	5 (5)	27 (27)				
**Includes search from PubMed**						0.06 (1,1)		.81
	No	36 (36)	17 (17)	53 (53)				
	Yes	33 (33)	14 (14)	47 (47)				
**Includes search from Web of Science**						0.39 (1,1)		.53
	No	53 (53)	22 (22)	75 (75)				
	Yes	16 (16)	9 (9)	25 (25)				
**Includes search from Cochrane**						0.15 (1,1)		.70
	No	35 (35)	17 (17)	52 (52)				
	Yes	34 (34)	14 (14)	48 (48)				
**Includes search from Trial Registries**						0.90 (1,1)		.34
	No	47 (47)	24 (24)	71 (71)				
	Yes	22 (22)	7 (7)	29 (29)				
**Includes search from Embase**						3.77 (1,1)		.05
	No	14 (14)	12 (12)	26 (26)				
	Yes	55 (55)	19 (19)	74 (74)				
**Systematic reviews of RCTs^a^, NRSIs^b^, or both**						1.66 (2,1)		.44
	RCTs only	12 (12)	4 (4)	16 (16)				
	NRSIs only	37 (37)	14 (14)	51 (51)				
	Both RCTs and NRSIs	20 (20)	13 (13)	33 (33)				
**Systematic review had a protocol**						5.78 (1,1)		.02
	No	41 (41)	26 (26)	67 (67)				
	Yes	28 (28)	5 (5)	33 (33)				

^a^RCT: randomized controlled trial.

^b^NRSI: nonrandomized studies of interventions.

## Discussion

We found that studies published in SPJs are commonly included as primary studies in SRs published in high-impact dermatology journals. SRs that contained a meta-analysis were less likely to have a primary study published in an SPJ, which is a promising finding, as research has shown that studies published in predatory journals are of lower quality [1,3]. Interestingly, SRs that registered a protocol were significantly less likely to include a primary study published in an SPJ. We suspect this finding may be because authors of SRs with registered protocols may have more diligence and time to confirm that sources of publications were not published in an SJP. In our sample, the majority of primary studies from SPJs were published in the *Indian Journal of Dermatology, Venereology, and Leprology*—which was removed from the DOAJ directory secondary to the journal failing to adhere to best practice [5]. Although considered to be an SPJ, this journal’s articles are included in Embase and PubMed searches. Interestingly, 83% (44/53) of the studies published in SPJs were PubMed indexed. One way through which studies published in SPJs can obtain PubMed indexing is “backdoor publishing” via PubMed Central or the National Center for Biotechnology Information Bookshelf [7]. Currently, there is little direction on how to best manage SPJs; however, the consensus is that studies published in SPJs should be omitted because of their potential impact on data synthesis. Due to their potential threat to SRs and scientific evidence, we recommend that authors of SRs verify their primary studies by using Beall’s List and the DOAJ directory—a recommendation proposed by other studies exploring ways to minimize the inclusion of studies published in SPJs in SRs [8,9].

Our study’s limitations include only searching SRs using PubMed and only using Beall’s List and DOAJ lists of questionable journals. Additionally, authors of SRs included in this study may have unknowingly included an SPJ, as some SPJs were added to Beall’s List and the DOAJ lists of questionable journals after the SR was already published, which is another limitation of our study. Lastly, future research is needed to investigate the effects of including SPJ publications in scientific research.

## Abbreviations

DOAJ: Directory of Open Access Journals
SPJ: suspected predatory journal
SR: systematic review
